# Glycogen Levels in Undiluted Genital Fluid and Their Relationship to Vaginal pH, Estrogen, and Progesterone

**DOI:** 10.1371/journal.pone.0153553

**Published:** 2016-04-19

**Authors:** Paria Mirmonsef, Anna L. Hotton, Douglas Gilbert, Casey J. Gioia, Danijela Maric, Thomas J. Hope, Alan L. Landay, Gregory T. Spear

**Affiliations:** 1 Department of Immunology/Microbiology, Rush University Medical Center, Chicago, Illinois, United States of America; 2 The CORE Center, Cook County Health & Hospital System, Chicago, Illinois, United States of America; 3 Robert H. Lurie Medical Research Center, Northwestern University Feinberg School of Medicine, Chicago, Illinois, United States of America; Fred Hutchinson Cancer Center, UNITED STATES

## Abstract

**Background:**

Colonization of the female lower genital tract with *Lactobacillus* provides protection against STIs and adverse pregnancy outcomes. Growth of genital *Lactobacillus* is postulated to depend on epithelial cell-produced glycogen. However, the amount of cell-free glycogen in genital fluid available for utilization by *Lactobacillus* is not known.

**Methods:**

Eighty-five genital fluid samples from 7 pre-menopausal women taken over 4–6 weeks were obtained using the Instead SoftCup^®^ (EvoFem, Inc., San Diego, CA, USA) by consented donors. Cell-free glycogen and glucose in genital fluids and estrogen and progesterone in blood were quantified.

**Findings:**

Glycogen ranged from 0.1–32 μg/μl. There were significant differences between women in glycogen over the observation period. There was a strong negative correlation between glycogen and vaginal pH (r = -0.542, p<0.0001). In multivariable analysis, free glycogen levels were significantly negatively associated with both vaginal pH and progesterone (p < 0.001 and p = 0.004, respectively). Estrogen, glucose, age, sexual intercourse 24 hours prior to visit, and days after the initial visit were not significantly associated with free glycogen levels.

**Conclusion:**

Cell-free glycogen concentrations can be very high, up to 3% of genital fluid, and are strongly associated with acidic vaginal pH. However, the fluctuations in glycogen levels in individuals and differences between individuals do not appear to be associated with estrogen.

## Introduction

A lower genital tract microbiota that is predominantly comprised of bacteria of the genus *Lactobacillus* is considered to be protective from acquiring a number of sexually transmitted infections (STIs) [1–3]. Also, *Lactobacillus* protects against acquisition of bacterial vaginosis [4–6], which while not an STI is a risk factor for premature labor and other pregnancy complications. An important mediator of this protection is thought to be the acidic pH of the lower genital tract resulting from production of lactic acid by *Lactobacillus* [7]. The vaginal pH of women with a genital microbiota dominated by *Lactobacillus* species is on average lower than 4.5 and recent studies indicate the pH may reach as low as <3.5 [8–10]. Besides a low pH, *Lactobacillus* may also provide protection via other mechanisms such as production of bacteriocins [11].

Glycogen is produced by vaginal and cervical epithelial cells and has been recognized as temporally associated with colonization and growth of *Lactobacillus* in the lower genital tract [12]. Thus, when epithelial glycogen is low, as in pre-puberty and post-menopause, *Lactobacillus* levels are also low [12]. Also, treatment with DMPA (Depo-Provera; depot medroxyprogesterone acetate) reduces the thickness of the glycogen bearing layers of cells in the epithelium and concurrently reduces the numbers of H_2_O_2_-positive *Lactobacillus* [13].

Estrogen has been postulated to induce glycogen production within the epithelium since it is also lower during the stages of life when glycogen is low, and lower during treatment with DMPA [13]. Further, several studies showed that administration of estrogen decreased vaginal pH (Reviewed in [14]).

While glycogen is present in the genital epithelium, *Lactobacillus* grows and metabolizes in the lumen of the vagina and not in epithelial cells. Therefore it is likely that the amount of cell-free glycogen that is present in vaginal fluid is an important factor in *Lactobacillus* growth and acid production. However, levels of cell-free glycogen in genital fluid are not determined in most studies of factors that affect genital *Lactobacillus*. Recently, two studies quantified the amount of cell-free glycogen in vaginal fluid and compared these values to vaginal pH and microbiota. In the first study [15], cervico-vaginal lavage (CVL) samples were collected from 21 African American women over an 8–11 year period. Samples with the highest cell-free glycogen had a lower genital pH and a higher fraction of the microbiota consisting of *Lactobacillus* as determined by pyrosequencing of the 16S rRNA gene. The second study measured cell-free glycogen over a 1–3 month period in 11 premenopausal and 12 postmenopausal women [16]. Although cell-free glycogen was observed in samples from both groups, premenopausal women had higher glycogen levels. In both groups, glycogen levels correlated with an acidic vaginal pH and *Lactobacillus* levels as quantified by real-time PCR. In those two studies, genital fluid was collected by lavage with 10 ml of saline. Therefore, the original concentration of glycogen in the genital fluid could not be determined in those studies since the original volume of genital fluid in women can vary [17]. Also, the method of collecting genital fluid by lavage could have artificially contributed to some of the observed variations seen between or within individuals and made correlations unreliable.

In this study, we obtained undiluted genital fluid from women using the Instead SoftCup^®^ so that possible variations due to lavage could be avoided and the concentration of cell-free glycogen present in the vagina could be measured. This enabled determining the levels of glycogen that are available for utilization by genital bacteria and quantifying true variations in glycogen levels over time. Additionally, collection of undiluted genital fluid allowed measurement of glucose, which is present at relatively low levels in genital fluid. Further, the relationships between glycogen and estrogen, progesterone, and vaginal pH were determined.

## Methods

### Ethics Statement

The Institutional Review Boards of Rush University Medical Center and Northwestern University approved the study and written informed consent was obtained from all subjects.

### Patients And Sample Acquisition

Women self-reported a regular menstrual cycle (between 25–30 days) and any hormonal birth control usage during the month prior to study entry. As part of routine visits, subjects were also asked to optionally divulge sexual activity information in the 24 hours prior to sample collection and if any type of barrier protection was utilized during intercourse. Women on hormonal birth control, those with irregular menstrual cycles, and those with intrauterine birth control devices were excluded from the study.

Cervical vaginal fluid samples were obtained from participants utilizing the Instead SoftCup^®^, and an average of 14 samples (a minimum of 9 and a maximum 18) were collected from each person over a 6-week period. The flexible cup was worn internally around the cervix and collected lower genital tract secretions. Subjects self-inserted the cup into their vagina at least 3 hours prior to their scheduled research visit. On arrival, subjects were instructed to manually remove the cup and place the entire apparatus into the provided 50 ml conical tube. The tube was capped and placed in a storage bag for transport to the lab at room temperature. Samples arrived in the lab approximately 10–20 minutes after they were obtained from the subject and were refrigerated until processed.

Within 24 hours of acquisition, the 50 ml conical tube containing the cup was centrifuged at 4°C, 800g for 10 minutes. The Instead SoftCup^®^ was then removed from the tube using tweezers and discarded, leaving fluid at the bottom of the tube. The top layer of fluid was pipetted into a 1.5 ml Eppendorf tube, and labeled. The pH was then measured utilizing a pH probe that was sprayed with ethanol, rinsed with distilled water and air dried before insertion.

Subjects also had two 4 ml tubes of blood drawn, once a week for 6 weeks, to measure serum levels of progesterone and estradiol by ELISA (Quest Diagnostics, Madison, NJ).

### Glycogen and Glucose Measurement

Free glycogen in genital fluid samples was measured fluorometrically using the Glycogen Assay Kit (BioVision, Milpitas, CA). Briefly, 10 μl of genital fluid or saline (serving as blank) was added to test wells in a 96-well plate. The volume was adjusted to 50 μl with hydrolysis buffer, with (total) or without (glucose background) the kit hydrolysis enzyme. Glucose background (samples without hydrolysis enzyme) was subtracted to determine glycogen concentration. The Relative Fluorescence Units (RFU) were measured using a BioTek Synergy HT plate reader (BioTek Instruments, Inc, Winooski, VT). Samples were diluted in 1X PBS (1:25, 1:50, 1:200, and 1:400) and glycogen was measured for each dilution in duplicate assays. The intra-assay coefficient of variation (CV) was between 10–13% All samples from one subject were run in one assay to avoid batch-to-batch changes in the assay.

### Statistical Analysis

Exploratory statistical analysis was performed using the Instat statistical software package (GraphPad Software) and regression analyses were performed using SAS software version 9.3 (SAS Institute, Cary, NC). Nonparametric methods were used for comparisons and to assess correlations, as indicated in the figure legends. A p-value of 0.05 or lower was considered significant.

A natural log transformation was applied to the distributions of estrogen, progesterone, and glycogen concentration to normalize the distributions and improve model fit. Mixed effects linear regression models with random intercept were used to examine univariable and multivariable associations of predictor variables with log glycogen concentration, accounting for correlation of repeated measures of individuals over time. Assumptions of linear regression models were assessed using standard regression diagnostics, including graphical examination of residual and normal probability plots and comparison of observed and predicted values, and were found to indicate adequate model fit. Sensitivity analyses were conducted to assess different treatment of undetectable concentrations (i.e., setting to missing values to 0 and excluding missing values) and yielded similar findings. Therefore, undetectable glucose and glycogen concentrations were set to 0 in the models. Spearman rank order correlation coefficients were calculated among all study variables to assess potential for multicollinearity. All variables of interest (age, estrogen and progesterone levels, glucose levels, pH, sex in the past 24 hours and time) were initially assessed in univariable analysis, and variables with p<0.2 in univariable analysis were entered in a multivariable regression model. Time in days from the index visit was included in all models to assess fluctuations in glycogen concentration over time.

## Results

### Longitudinal cell-free glycogen and glucose levels in undiluted vaginal fluid

Since glycogen levels in undiluted lower genital tract fluid have not been described, we measured free glycogen levels in 85 undiluted vaginal fluid samples collected from 7 pre-menopausal women over 4–6 weeks. The subjects’ demographics are shown in Table 1.

**Table 1:** Subjects’ characteristics at index visit, n = 7.

	n (%) or median (range)
**Age (years)**	30 (26–48)
**Race/Ethnicity**	
White	1 (14.3)
Black	5 (71.4)
Hispanic	1 (14.3)
**Sex in last 24 hours**	1 (14.3)
**pH**	5.1 (3.1–7.8)
**Estrogen (pg/ml)**	134 (50–527)
**Progesterone (ng/ml)**	1.89 (0.19–6.54)
**Glycogen (μg/μl)**	0.76 (0.11–19.8)
**Glucose (μg/μl)**	0.0 (0.0–0.0)

**Fig 1:**
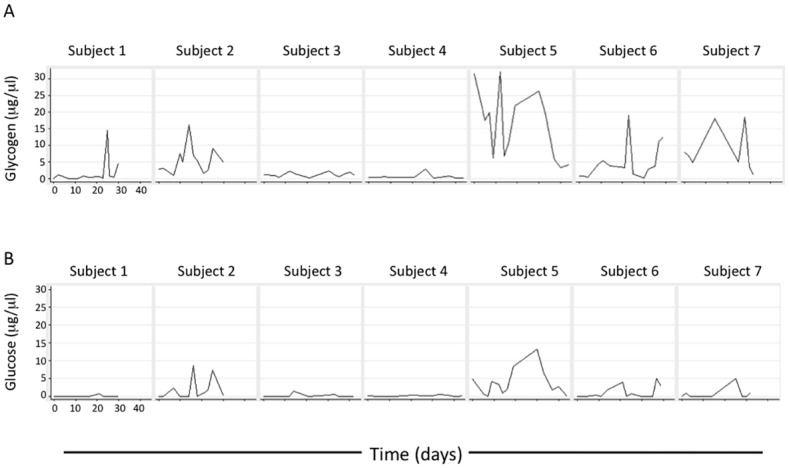
Longitudinal cell-free glycogen and glucose in undiluted vaginal secretions. Vaginal fluid was collected from seven pre-menopausal subjects using the Instead SoftCup^®^ as described in Methods. Glycogen (A) and glucose (B) levels were measured in undiluted vaginal fluid.

The glycogen median and mean ± SD for all samples was 2.8 μg/μl and 5.5 ± 7.4 μg/μl with a range of undetectable (<0.1) to 32 μg/μl (Fig 1A). Subject 5 had the highest glycogen levels over the sampling period with median and mean values of 17.6 μg/μl and 15.9 μg/μl, respectively.

While levels of glycogen fluctuated for each woman over the observation period, there were significant differences in overall glycogen levels between some of the women. Thus, subject 5 had significantly (Kruskal-Wallis Dunn’s test) higher levels of glycogen over the observation period than subjects 1, 3 and 4, but not 2, 6 and 7. Subject 7, who had the second highest overall levels of glycogen (median of 6.0 μg/μμl, mean of 8.3 μg/μl) had significantly higher levels than subjects 1 and 4 but not 2, 3, 5 and 6. Subject 2 had the third highest levels of glycogen (median of 5.0 μg/μl, mean of 5.8 μg/μl) and was significantly higher than subjects 1 and 4 but not the others.

The median and mean ± SD for all glucose observations were undetectable (<0.1 μg/μl) and 1.2 ± 2.4 μg/μl and ranged from undetectable to 13.3 μg/μl. Subject 5 had significantly higher glucose overall than subjects 1, 3 and 4 (Kruskal-Wallis Dunn’s test). No other comparisons were significant. There was a highly significant positive correlation between glucose and glycogen (Spearman r = -0.4102, p<0.0001, S1A Fig), and a negative correlation between pH and glycogen (Spearman r = -0.5418, p<0.0001, S1B Fig). There was also a small negative, albeit significant, correlation between progesterone and glycogen (Spearman r = -0.34, p = 0.0368, S1C Fig). Plasma estrogen levels and glycogen did not significantly correlate (Spearman r = -0.2479, p = 0.1574, S1D Fig).

### Longitudinal vaginal pH, serum estrogen and progesterone

**Fig 2:**
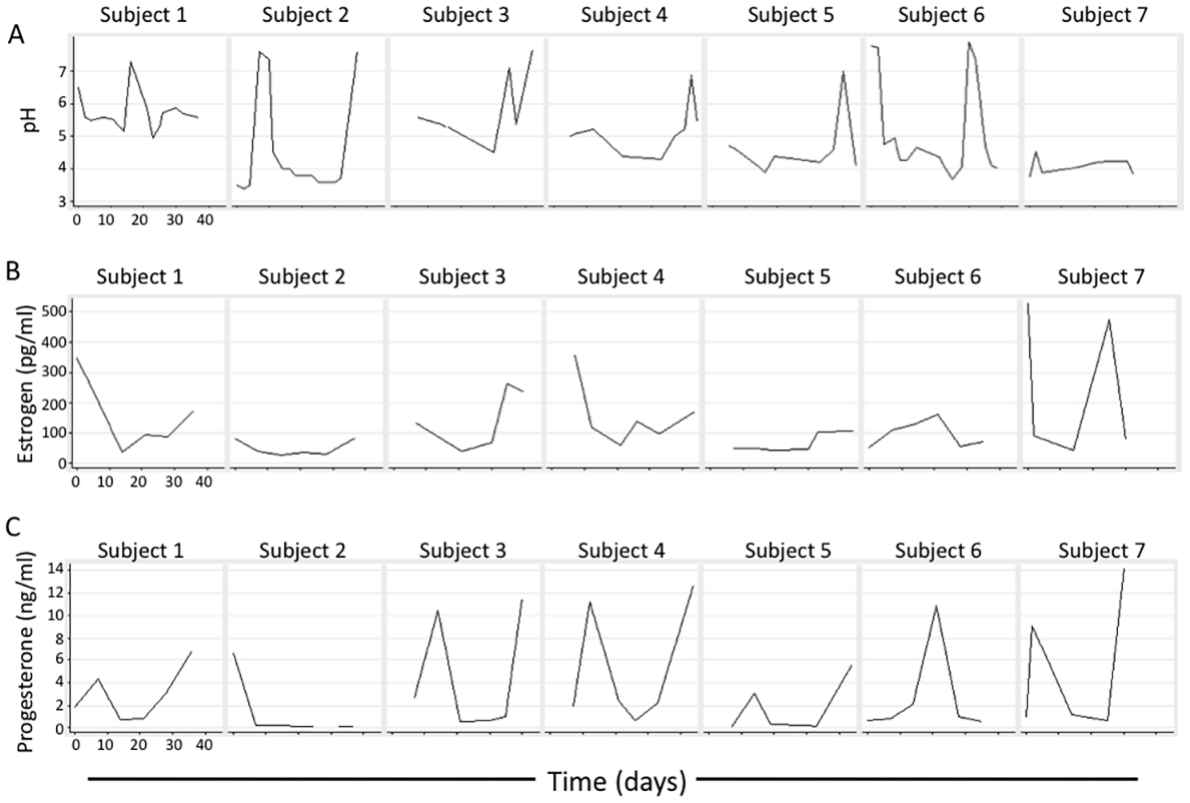
Longitudinal vaginal pH, serum estrogen and progesterone. Vaginal pH (A) was determined utilizing a sterile pH probe as described in Methods. Blood was drawn from the seven subjects shown in Fig 1. Serum levels of estrogen (B) and progesterone (C) were measured by ELISA.

Vaginal pH was measured at 79 of the visits as an indicator of the bacterial microbiota (Fig 2A). A vaginal pH cutoff of 4.5 is used clinically with a pH greater than 4.5 indicative of dysbiosis [8]. pH levels overall (N = 79) ranged from 3.4 to 7.9 with a median of 4.6 and mean ± SD of 5.0 ± 1.2. Visits with a pH < 4.5 had significantly higher levels of glycogen than visits with a pH ≥ 4.5 (median 5.2 μg/μl vs. 0.8 μg/μl, p = 0.0001, Mann-Whitney test). Also, there was a relatively strong negative correlation between pH and glycogen (N = 67, r = -0.542, p < 0.0001).

Since it is proposed that estrogen promotes glycogen expression in lower genital tract epithelium we examined if serum estrogen concentrations corresponded to levels of free glycogen levels in undiluted vaginal fluid. As expected, estrogen and progesterone levels varied over time in each woman (Fig 2B and 2C). However there was no significant correlation between glycogen and estrogen (Spearman r = -0.2, p > 0.05, n = 35). There was a significant negative association of glycogen with progesterone (p = 0.037, Spearman r = -0.349, n = 36).

A multivariable analysis was performed to further analyze the factors associated with glycogen. For this analysis, there were 29 time points where all data, including estrogen, progesterone, glucose, pH and sexual activity were available. Using this data set in univariable analysis, vaginal pH was again significantly negatively associated with glycogen concentration (Table 2). There was a trend towards an association between glycogen and estrogen, although this was a negative association.

**Table 2:** Analysis of factors associated with glycogen concentration (n = 29).

	Univariable Effect Estimate^a^ (95% CI)	*P*	Multivariable Effect Estimate^a^ (95% CI)	*P*
Age (years)	0.07 (-0.05, 0.20)	0.248	--	--
Estrogen (natural log)	-0.40 (-0.88, 0.07)	0.089	-0.32 (-0.65, 0.01)	0.057
Progesterone (natural log)	-0.25 (-0.52, 0.01)	0.062	-0.30 (-0.49, -0.11)	0.004
Glucose (μg/μl)	0.09 (-0.08, 0.27)	0.270	--	--
pH	-0.49 (-0.78, -0.19)	0.003	-0.51 (-0.75, -0.27)	<0.001
Sex in last 24 hours	-0.95 (-2.01, 0.12)	0.079	--	--
Time (days)	0.0004 (-0.03, 0.03)	0.974	-0.01 (-0.03, 0.01)	0.337

Results from Mixed Effects Regression Models.

^a^. Multivariable model is adjusted for variables for which estimates are presented. Estimates are generated from linear mixed effects model with random intercept and represent the change in log glycogen concentration per unit increase in the exposure of interest. CI = confidence interval

In multivariable analysis, which controlled for estrogen, progesterone and time in days since the initial visit, pH was again significantly negatively associated with glycogen (p < 0.001, Table 2). Progesterone was also significantly negatively associated with glycogen (p = 0.004), while there was a trend for a negative association with estrogen. Glucose levels, age, sexual intercourse 24 hours prior to visit, and days after the initial visit were not significantly associated with glycogen.

## Discussion

This study obtained undiluted genital fluid from women so that levels of cell-free glycogen could be accurately measured. We found that when evaluating all samples, there was a very large range of glycogen levels, from undetectable to 32 μg/μl. Some of this broad range in levels appeared to be explained by significant person-to-person differences; for example, subject 5 had median glycogen levels of 17.6 μg/μl while subject 4 had median levels of 0.4 μg/μl, a 44-fold difference. However, even within women, there were relatively large fluctuations in glycogen over the relatively short period of sample acquisition. Thus, the highest levels for subject 5 was 32 μg/μl while the lowest level for this subject was 3.4 μg/μl. This represents nearly a 10-fold difference from the highest to lowest levels over the relatively short collection period.

Some large person-to-person variations and within-person variations were also seen in our previous studies of cell-free glycogen where samples were collected by lavage [15, 16]. However, there was a concern that the lavage sampling method could have artificially caused some of those variations since the amount of genital fluid can vary [17]. Since the current study obtained undiluted fluid, this provides evidence that the relatively rapid and large variations seen in individuals and large differences between individuals are real.

Interestingly, this study did not show a positive correlation between estrogen and cell-free glycogen, when analyzed in univariate or multivariate models. A previous study using lavage also did not show a significant relationship between estrogen and glycogen [16]. However, there was a significant negative correlation between progesterone and glycogen in the multivariate model. It has been established that high doses of progesterone reduce epithelial thickness [13, 18, 19] and it is possible the natural increases in progesterone that occur during the menstrual cycle could have subtle effects on shedding of glycogen-containing epithelial cells thus resulting in lower free glycogen in genital fluid. In fact, a small reduction in epithelial thickness was seen in the later stages of the menstrual cycle [20].

Alternatively, if estrogen does indeed induce glycogen accumulation in genital epithelium and changes in estrogen during the menstrual cycle affect the amount of glycogen in epithelium, we speculate that there would be a lag time for that change in glycogen to be observed in cell-free soluble glycogen in the vaginal lumen. This lag would be due to the time it would take for the epithelial layers that are most metabolically active (presumably the more basal layers) to differentiate, move up the layers and be shed or release glycogen. Since this time may take at least several days, a lack of correlation between concurrent blood estrogen and cell-free glycogen might be expected. This lag might also help explain the significant but negative correlation between glycogen and progesterone. However, one would expect that women that have chronically low estrogen would have overall lower glycogen levels (there is substantial variation in population). However, in our study, subjects 2 and 5 with some of the lowest overall estrogen levels had some of the highest overall glycogen levels. An explanation for this is currently unapparent.

Some of the evidence for a close relationship between estrogen, glycogen and *Lactobacillus* colonization has been the observation that around the onset of puberty the lower genital tract typically becomes colonized with *Lactobacillus* species and vaginal pH decreases. However, in a recent study of 31 premenarcheal girls, Hickey et. al found that lactobacilli colonization can occur at least a year prior to menarche [21]. *Lactobacillus* colonization in these girls was not necessarily concurrent with a decrease in pH, perhaps because of low bacterial loads. It will be interesting to study hormone levels in this population to assess their relationship with glycogen and *Lactobacillus* colonization.

This study also found a significant negative relationship between cell-free glycogen levels in undiluted genital fluid and genital pH. The previous studies that used lavage also found this relationship, but using undiluted fluids confirms this association. Since most of the acidity in genital fluid is due to lactic acid produced by *Lactobacillus*, the current study provides additional evidence to support the hypothesis that cell-free glycogen provides a carbohydrate substrate for growth of *Lactobacillus* in the genital tract. Culture media used to grow *Lactobacillus* generally contains 2% glucose and *Lactobacillus* can grow fairly rapidly even in 1% glucose [22]. We observed cell-free glycogen as high as 3%. Thus, at many of the time points, the amount of carbohydrate, in the form of glycogen, is high enough to sustain growth of *Lactobacillus*.

However, a number of studies showed that lactobacilli do not directly utilize glycogen in medium when it is the only source of carbohydrates [23–25]. A recent study showed that glycogen is broken down into dimers (maltose), trimers and tetramers of glucose by alpha-amylase that is present in genital tract secretions [22]. An important unanswered question is how much of the breakdown products are present in genital secretions and how do they correspond to glycogen and *Lactobacillus* levels. In our study, we did not assay those breakdown products due to the large amount of genital fluid needed for the high-performance anion-exchange chromatography method.

This study also showed there is measurable glucose in genital secretions. Generally, glucose is present at much lower concentrations than glycogen (Fig 1) and the use of undiluted genital fluid facilitated glucose measurement in our study. In univariable analysis using all available data there was a strong, significant correlation between glucose and glycogen suggesting that glycogen was the source of glucose. A plot of glucose versus glycogen (data not shown) showed that many fluids had detectable glycogen and glucose, while some fluids had detectable glycogen but undetectable glucose. However, there were no fluids that had detectable glucose with no detected glycogen. This further supports the hypothesis that the source of glucose was breakdown of glycogen.

Several recent studies showed that enzymes that can break down glycogen into glucose are present in the genital secretions of some women [22, 26]. We hypothesize that those host-derived enzymes are the source of much of the glucose although it is possible that microbial enzymes could also contribute. Further studies are needed to determine the relative amounts of glucose monomers, dimers, trimers and tetramers in genital fluid and how these levels relate to the types of microbiota present. It is intriguing to speculate that different ratios of these multimers or excess glucose generation from glycogen could impact the makeup of the microbiota.

There were several limitations to this study. This study included a relatively small number of participants. Information on sexual behaviors, symptoms, genital co-infections, stage of menstrual cycle, and use of vaginal products was not available. This could have limited our ability to make inferences about the mechanisms underlying observed associations. It would have also been of interest to identify the vaginal microbiota in these samples and assess their relationship with glycogen levels. Moreover, while ELISA was used to measure sex hormones, the combined use of ELISA and mass spectrometry may more accurately determine the concentration of estrogen and progesterone [27].

Altogether, our results show that cell-free glycogen concentrations can be very high in genital fluid, can vary substantially between women and over time in the same woman, and point to the complexity of the relationship between sex hormones and glycogen. Further studies of factors that impact cell-free glycogen levels in vaginal fluids are needed since cell-free glycogen appears to be strongly related to genital microbiota type.

## Supporting Information

S1 Fig Relationships between free glycogen and glucose, pH, progesterone, and estrogen. Spearman correlations between glycogen and glucose (A), pH (B), progesterone (C), and estrogen (D) were calculated using samples that exhibited values for both x and y.
(XLSX)

## References

[1] Cherpes TL, Meyn LA, Krohn MA, Lurie JG, Hillier SL. Association between acquisition of herpes simplex virus type 2 in women and bacterial vaginosis. Clin Infect Dis. 2003;37(3):319–25. . 12884154 10.1086/375819

[2] Wiesenfeld HC, Hillier SL, Krohn MA, Landers DV, Sweet RL. Bacterial vaginosis is a strong predictor of Neisseria gonorrhoeae and Chlamydia trachomatis infection. Clin Infect Dis. 2003;36(5):663–8. . 12594649 10.1086/367658

[3] Atashili J, Poole C, Ndumbe PM, Adimora AA, Smith JS. Bacterial vaginosis and HIV acquisition: a meta-analysis of published studies. AIDS. 2008;22(12):1493–501. Epub 2008/07/11. [pii]. . 18614873 10.1097/QAD.0b013e3283021a37 PMC2788489

[4] Hawes SE, Hillier SL, Benedetti J, Stevens CE, Koutsky LA, Wolner-Hanssen P, et al Hydrogen peroxide-producing lactobacilli and acquisition of vaginal infections. J Infect Dis. 1996;174(5):1058–63. . 8896509 10.1093/infdis/174.5.1058

[5] Cherpes TL, Hillier SL, Meyn LA, Busch JL, Krohn MA. A delicate balance: risk factors for acquisition of bacterial vaginosis include sexual activity, absence of hydrogen peroxide-producing lactobacilli, black race, and positive herpes simplex virus type 2 serology. Sex Transm Dis. 2008;35(1):78–83. Epub 2007/11/09. . 17989585 10.1097/OLQ.0b013e318156a5d0

[6] Vallor AC, Antonio MA, Hawes SE, Hillier SL. Factors associated with acquisition of, or persistent colonization by, vaginal lactobacilli: role of hydrogen peroxide production. J Infect Dis. 2001;184(11):1431–6. Epub 2001/12/26. JID010451 [pii] 10.1086/324445 . 11709785

[7] Boskey ER, Telsch KM, Whaley KJ, Moench TR, Cone RA. Acid production by vaginal flora in vitro is consistent with the rate and extent of vaginal acidification. Infect Immun. 1999;67(10):5170–5. Epub 1999/09/25. 10496892 10.1128/iai.67.10.5170-5175.1999 PMC96867

[8] Amsel R, Totten PA, Spiegel CA, Chen KC, Eschenbach D, Holmes KK. Nonspecific vaginitis. Diagnostic criteria and microbial and epidemiologic associations. Am J Med. 1983;74(1):14–22. 6600371 10.1016/0002-9343(83)91112-9

[9] O'Hanlon DE, Moench TR, Cone RA. Vaginal pH and microbicidal lactic acid when lactobacilli dominate the microbiota. PLoS ONE. 2013;8(11):e80074 Epub 2013/11/14. [pii]. 24223212 10.1371/journal.pone.0080074 PMC3819307

[10] Ravel J, Gajer P, Abdo Z, Schneider GM, Koenig SS, McCulle SL, et al Microbes and Health Sackler Colloquium: Vaginal microbiome of reproductive-age women. Proc Natl Acad Sci U S A. 2010;108 Suppl1:4680–7. Epub 2010/06/11. 1002611107 [pii]. 20534435 10.1073/pnas.1002611107 PMC3063603

[11] Mirmonsef P, Spear GT. The Barrier to HIV Transmission Provided by Genital Tract Lactobacillus Colonization. Am J Reprod Immunol. 2014;71:531–6. Epub 2014/03/26. 10.1111/aji.12232 . 24661438

[12] Cruickshank R, Sharman A. The biology of the vagina in the human subject. II. The bacterial flora and secretion of the vagina at various age-periods and their relation to glycogen in the vagind epithelium. J Obstet Gynaec Brit Emp. 1934;41:208.

[13] Miller L, Patton DL, Meier A, Thwin SS, Hooton TM, Eschenbach DA. Depomedroxyprogesterone-induced hypoestrogenism and changes in vaginal flora and epithelium. Obstet Gynecol. 2000;96(3):431–9. Epub 2000/08/29. S0029-7844(00)00906-6 [pii]. . 10960638 10.1016/s0029-7844(00)00906-6

[14] Krause M, Wheeler TL 2nd, Snyder TE, Richter HE. Local Effects of Vaginally Administered Estrogen Therapy: A Review. J Pelvic Med Surg. 2009;15(3):105–14. 10.1097/SPV.0b013e3181ab4804 22229022 PMC3252029

[15] Mirmonsef P, Hotton AL, Gilbert D, Burgad D, Landay A, Weber KM, et al Free Glycogen in Vaginal Fluids Is Associated with Lactobacillus Colonization and Low Vaginal pH. PLoS ONE. 2014;9(7):e102467 Epub 2014/07/18. 10.1371/journal.pone.0102467 . 25033265 PMC4102502

[16] Mirmonsef P, Modur S, Burgad D, Gilbert D, Golub ET, French AL, et al Exploratory comparison of vaginal glycogen and Lactobacillus levels in premenopausal and postmenopausal women. Menopause. 2014;22:1–8. Epub 2014/12/24. 10.1097/GME.0000000000000397 . 25535963 PMC4476965

[17] Dezzutti CS, Hendrix CW, Marrazzo JM, Pan Z, Wang L, Louissaint N, et al Performance of swabs, lavage, and diluents to quantify biomarkers of female genital tract soluble mucosal mediators. PLoS ONE. 2011;6(8):e23136 10.1371/journal.pone.0023136 21858008 PMC3155537

[18] Marx PA, Spira AI, Gettie A, Dailey PJ, Veazey RS, Lackner AA, et al Progesterone implants enhance SIV vaginal transmission and early virus load. Nat Med. 1996;2(10):1084–9. Epub 1996/10/01. . 8837605 10.1038/nm1096-1084

[19] Hild-Petito S, Veazey RS, Larner JM, Reel JR, Blye RP. Effects of two progestin-only contraceptives, Depo-Provera and Norplant-II, on the vaginal epithelium of rhesus monkeys. AIDS Res Hum Retroviruses. 1998;14 Suppl 1:S125–30. Epub 1998/05/15. . 9581896

[20] Patton DL, Thwin SS, Meier A, Hooton TM, Stapleton AE, Eschenbach DA. Epithelial cell layer thickness and immune cell populations in the normal human vagina at different stages of the menstrual cycle. Am J Obstet Gynecol. 2000;183(4):967–73. Epub 2000/10/18. S0002-9378(00)44304-8 [pii] 10.1067/mob.2000.108857 . 11035348

[21] Hickey RJ, Zhou X, Settles ML, Erb J, Malone K, Hansmann MA, et al Vaginal microbiota of adolescent girls prior to the onset of menarche resemble those of reproductive-age women. MBio. 2015;6(2). Epub 2015/03/26. mBio.00097-15 [pii] 10.1128/mBio.00097-15 25805726 PMC4453513

[22] Spear GT, French AL, Gilbert D, Zariffard MR, Mirmonsef P, Sullivan TH, et al Human alpha-amylase present in lower genital tract mucosal fluid processes glycogen to support vaginal colonization by Lactobacillus. J Infect Dis. 2014;210:1019–28. Epub 2014/04/17. 10.1093/infdis/jiu231 . 24737800 PMC4168305

[23] Wylie JG, Henderson A. Identity and glycogen-fermenting ability of lactobacilli isolated from the vagina of pregnant women. J Med Microbiol. 1969;2(3):363–6. Epub 1969/08/01. . 4996481 10.1099/00222615-2-3-363

[24] Martin R, Soberon N, Vaneechoutte M, Florez AB, Vazquez F, Suarez JE. Characterization of indigenous vaginal lactobacilli from healthy women as probiotic candidates. Int Microbiol. 2008;11(4):261–6. Epub 2009/02/11. im2306110 [pii]. . 19204898 10.2436/20.1501.01.70

[25] Stewart-Tull DE. Evidence That Vaginal Lactobacilli Do Not Ferment Glycogen. Am J Obstet Gynecol. 1964;88:676–9. Epub 1964/03/01. . 14128200 10.1016/0002-9378(64)90898-1

[26] Spear GT, McKenna M, Landay AL, Makinde H, Hamaker B, French AL, et al Effect of pH on Cleavage of Glycogen by Vaginal Enzymes. PLoS ONE. 2015;10(7):e0132646 10.1371/journal.pone.0132646 26171967 PMC4501710

[27] Zendjabil M CZ, Abbou O. Role of mass spectrometry in steroid assays. Ann Endocrinol (Paris). 2016;77(1). Epub 2016 Feb 9. 10.1016/j.ando.2016.01.004 26872617

